# Metallurgical Preparation of Nb–Al and W–Al Intermetallic Compounds and Characterization of Their Microstructure and Phase Transformations by DTA Technique

**DOI:** 10.3390/molecules25082001

**Published:** 2020-04-24

**Authors:** Tomas Cegan, Daniel Petlak, Katerina Skotnicova, Jan Jurica, Bedrich Smetana, Simona Zla

**Affiliations:** VSB—Technical University of Ostrava, Faculty of Materials Science and Technology, 70800 Ostrava, Czech Republic; daniel.petlak@vsb.cz (D.P.); katerina.skotnicova@vsb.cz (K.S.); jan.jurica@vsb.cz (J.J.); bedrich.smetana@vsb.cz (B.S.); simona.zla@vsb.cz (S.Z.)

**Keywords:** intermetallics, niobium aluminide, tungsten aluminide, plasma arc melting, vacuum induction melting, microstructure, differential thermal analysis

## Abstract

The possibilities of metallurgical preparation of 40Nb-60Al and 15W-85Al intermetallic compounds (in at.%) by plasma arc melting (PAM) and vacuum induction melting (VIM) were studied. Both methods allow easy preparation of Nb–Al alloys; however, significant evaporation of Al was observed during the melting, which affected the resulting chemical composition. The preparation of W–Al alloys was more problematic because there was no complete re-melting of W during PAM and VIM. However, the combination of PAM and VIM allowed the preparation of W–Al alloy without any non-melted parts. The microstructure of Nb–Al alloys consisted of Nb_2_Al and NbAl_3_ intermetallic phases, and W–Al alloys consisted mainly of needle-like WAl_4_ intermetallic phase and Al matrix. The effects of melting conditions on chemical composition, homogeneity, and microstructure were determined. Differential thermal analysis was used to determine melting and phase transformation temperatures of the prepared alloys.

## 1. Introduction

Intermetallic compounds are defined as solid phases involving two or more metallic or semi-metallic elements with an ordered structure and often a well-defined and fixed stoichiometry. These compounds are characterized by a high melting point and an ordered nature, which imparts high specific strength at high temperature. These properties make them attractive candidates for many applications, especially in the aerospace and chemical industries. Some of the most promising are intermetallic compounds in the niobium–aluminium system [1,2]. These intermetallics are expected to be usable as structural materials at higher temperatures than conventional nickel-based superalloys because of their higher melting temperature and lower density [3]. However, binary Nb–Al intermetallics have low fracture toughness at ambient temperature. Many studies have been performed to see if these drawbacks can be overcome, especially with Nb_2_Al and NbAl_3_ [1,2]. Nb_2_Al is also seen as a superconducting material for the next generation [4]. NbAl_3_ has advantages in density and oxidation resistance [1,2]. One of the downsides of these attractive materials is that the processing is relatively difficult. Several methods have been tried—conventional melting and solidification, powder metallurgy, reaction sintering, reaction sintering with prior mechanical activation—but their preparation was mostly carried out in small volumes and weights [3,5,6,7,8]. Tungsten aluminide is an intermetallic material that has not been studied much. Binary compounds from the W–Al system show remarkable properties. Zhang et al. [9], for example, stated that tungsten aluminide and tungsten-reinforced aluminium matrix composites exhibit up to 50% higher hardness than pure Al. However, the low solubility of W in Al and the large difference between melting temperatures of W and Al make it difficult to fabricate W–Al intermetallic compounds. There are very few publications dealing with the preparation and properties of intermetallics based on W–Al, and powders are generally used as input materials, with subsequent processing by mechanical alloying, self-propagating high-temperature synthesis (SHS), or both [9,10,11,12]. Only limited information on the preparation of Nb–Al and W–Al alloys by conventional metallurgical methods is available. For all these reasons, we examined the preparation of Nb–Al and W–Al alloys by conventional metallurgy methods such as vacuum induction melting and plasma arc melting in this experiment. Plasma furnaces enable extremely high temperatures of the plasma arc to be achieved and sufficient performance allows melting of refractory metals [13]. Vacuum induction furnaces usually do not reach temperatures suitable for the melting of refractory metals; however, due to the high reactivity of melted Al, it is theoretically possible to prepare W–Al alloys by this method if the charge is optimised and the furnace and crucible are allowed longer stabilization times around temperatures 1600 °C and higher. Frkanova and Lapin [14] reported that it is possible to produce alloys with a composition of 14 at.% Ta and 86 at.% Al by vacuum induction melting in an Al_2_O_3_-based crucible with a stabilization time from 60–270 s at a temperature of about 1600 °C. The significant dissolution of W in the Al melt was also reported by Unuvar [11,12] during self-propagating high-temperature synthesis (SHS) at 1600 °C.

## 2. Results and Discussion

### 2.1. Chemical Composition and Homogeneity

#### 2.1.1. Nb–Al Alloys

**Figure 1:**
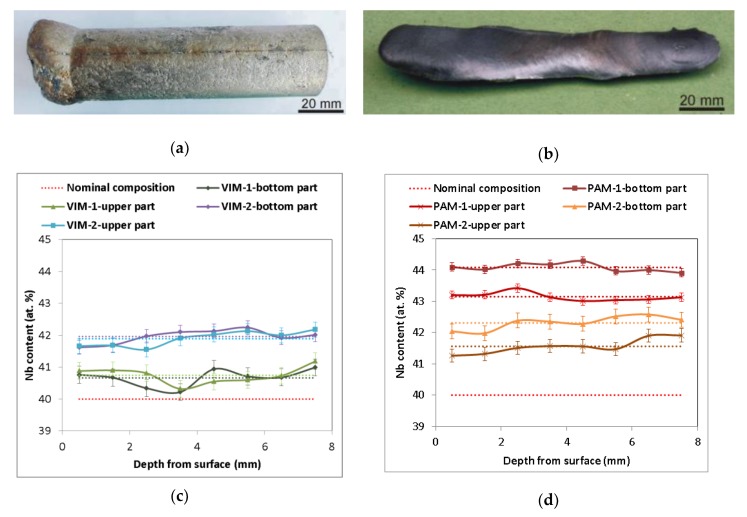
(**a**) Sample Nb–Al-VIM-1 after induction melting; (**b**) sample Nb–Al-PAM-1 after plasma arc melting; (**c**) concentration profile of cuts of Nb–Al samples after vacuum induction melting (VIM) (energy-dispersive X-ray spectrography (EDS), dotted lines indicate average value); (**d**) concentration profile of cuts of Nb–Al samples after plasma arc melting (PAM) (EDS, dotted lines indicate average value).

Figure 1a,b illustrates a typical example of a cylindrical ingot 28 mm in diameter and 90 mm long and of oval cross-section ingots about 8 mm and 18 mm in diameter and 140 mm long, prepared by VIM and PAM, respectively. A list of all prepared alloys including the method used is shown in Table 1. During all melting processes, Nb wires completely dissolved in the Al melt, as verified by the absence of insoluble residues of Nb wire inside the melting crucible after pouring and inside the castings. Figure 1c,d presents the chemical composition determined by EDS in individual sections of the prepared ingots. All Nb–Al alloy parts contained slightly higher amounts of Nb (average values from 40.7 at.% to 44.1 at.%) than the nominal composition (40 at.%). In every melted sample at a temperature of about 1400–1500 °C, a small amount of smoke started to escape from the melting crucible and condensed at the inner walls of the furnace and glass windows. An EDS analysis of condensed powder revealed a composition based on pure aluminium, which evaporated during melting, confirming the findings of other authors. A similar phenomenon was also described by Gomez and Yanging [15,16] during induction melting of γ-TiAl-based alloys from pure metals in cold crucibles or ceramic crucibles at similar temperatures and melting conditions, as well as by Blacha [17] during induction melting of a Ti-6Al-4V alloy. This phenomenon is related to the high vapor pressure of aluminium at the mentioned temperatures (about 100 Pa at 1600 °C) [18], which causes its evaporation accompanied by reduced content in the alloy. The same phenomenon is therefore probably responsible for the reduced aluminium content in Nb–Al alloys after VIM. The aluminium evaporation during VIM also indicates the results of EDS analysis shown in Figure 1c, which exhibit a more pronounced decrease in aluminium content in ingot VIM-2 (stabilization time 120 s) than VIM-1 (stabilization time 60 s). The aluminium loss after PAM is also related to the high vapor pressure of aluminium, especially with higher achieved temperatures during PAM, which can reach up to several thousand °C [13] (evaporation temperature of Al is 2519 °C). The aluminium evaporation during PAM also indicates the results of EDS analysis, which exhibit a more pronounced decrease in the aluminium content in ingot PAM-2 (four passes) than ingot PAM-1 (two passes). As can also be seen in the results of EDS analysis, the chemical composition was significantly affected by the method used. All measurements in transverse sections of Nb–Al alloys after PAM had relatively small deviations from average values (up to 0.35 at.%), but Nb content between the bottom and upper part was quite different (44.1 at.% and 43.2 at.% for PAM-1 and 42.3 at.% and 41.6 at.% for PAM-2, respectively). While all eight measurements in each transversal section after VIM had somewhat higher deviations from the average values (up to 0.44 at.%), Nb content between the bottom and upper part was almost the same (40.7 at.% and 40.8 at.% for VIM-1 and 42.0 at.% and 41.9 at.% for VIM-2, respectively), which indicates that the melt temperature and holding time of that temperature in both cases were sufficient to achieve nearly homogeneous chemical composition of all induction melted castings. Different chemical compositions in the bottom and upper parts of the ingots after plasma arc melting can be explained by the influence of phenomena typical for zone melting like Marangoni convection, segregation, and mass transfer connected with high temperature gradients during melting.

The contents of oxygen and nitrogen in the prepared intermetallic compounds were about 60 and 10 wt. ppm after PAM, respectively, and showed no significant differences with a number of passes. Oxygen content in prepared alloys was about 10 wt. ppm for VIM-1 and about 30 wt. ppm for VIM-2. Nitrogen content in the alloys showed values below 5 wt. ppm, which is the detection limit.

#### 2.1.2. W–Al Alloys

**Figure 2:**
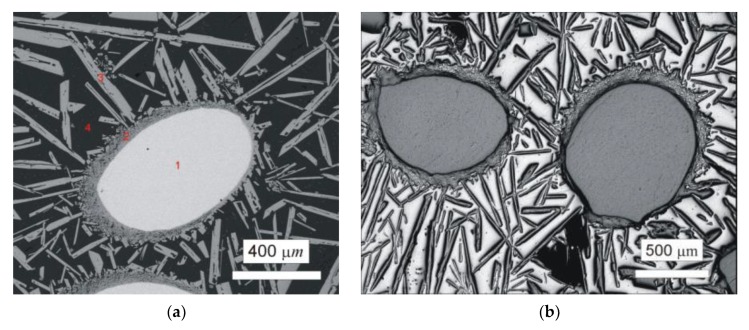
(**a**) Scanning electron microscopy back-scattered electron (SEM-BSE) image of the W–Al-PAM-1 alloy; (**b**) optical microscopy (OM) image of the W–Al-PAM-2-alloy.

First, plasma arc melting was carried out by quadruple passing of the plasma torch in the horizontal copper water-cooled crystallizer. It is important to note that significant evaporation of Al during plasma arc melting occurred and was much more pronounced than during the melting of Nb–Al alloys due to higher performance. Aluminium evaporation was also observed during induction melting of W–Al alloys. Figure 2a shows un-melted areas in the W–Al alloy prepared by the mentioned method. This area up to 1 mm^2^ was identified by EDS analysis as pure tungsten (see Table 2), suggesting that it failed to be melted due to the high melting point of tungsten. Around the areas of unmelted tungsten, a thin continuous layer of intermetallic phases based on tungsten and aluminium were present, and farther away from the tungsten were long needle-like particles of WAl_4_ phase in Al matrix. In order to avoid the persistence of un-melted areas, a new experiment of plasma arc melting with the same conditions and six passes was executed. Higher current density was not used because 800 A is the maximum current density for safety reasons. Figure 2b exhibits the microstructure after melting; as can be seen in this figure, the microstructure is almost the same and the unmelted areas are observed as well. The same results were obtained after the next two passes (total eight passes) under the same conditions, and the only significant difference was seen in the greater proportion of evaporated Al. The mentioned method is therefore unsuitable for the preparation of W–Al intermetallic compounds due to the existence of non-melted areas in the products that could not be removed even after multiple re-melting.

**Figure 3:**
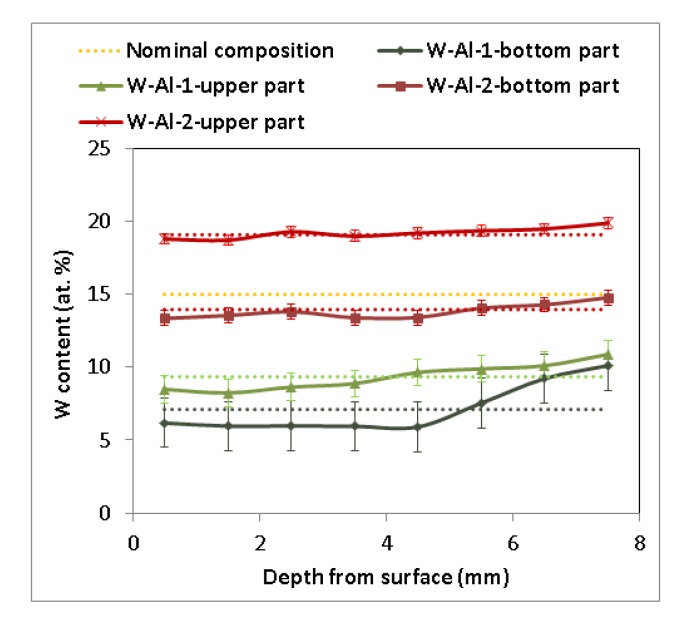
Concentration profile of cuts of W–Al samples after VIM (EDS, dotted lines indicate average value).

As another method, vacuum induction melting was tested with the use of 1 mm diameter W wires. It was evident that W wires did not completely dissolve in the Al melt, and insoluble residues of W wires inside the melting crucible were visible during the melting process and stabilization of the melt of W–Al alloys. After stabilization, the melt was poured into the copper mould. Several residues of W wires in the melting crucible were observed after pouring, but no W wires were observed inside the casting, indicating that all solid residues remained only on the bottom of the melting crucible. Figure 3 presents the chemical composition determined by EDS in individual sections of the prepared W–AlVIM-1 alloy ingot. The results of EDS analysis showed that the chemical composition of both parts of the W–Al-VIM-1 ingot contained considerably less W (7.1 at.% for the bottom part and 9.4 at.% for the upper part) than the nominal composition (15 at.% W) due to incomplete melting of W wires, but approximately half of the wire was dissolved in the aluminium melt. EDS analysis revealed significant differences between the bottom and upper parts of the ingot and with different depths from the surface of the mould. These differences are related to the solidification and formation of the microstructure and will be described in detail in the next subsection. In terms of chemical composition, the selected alloy with 15 at.% W was not prepared, but the alloy with 7–9 at.% W was. Because our aim was to prepare an alloy with a higher amount of W, another experiment was scheduled. This experiment initially consisted in preparing another 150 g ingot with the nominal composition of 20W–80Al (at.%) by plasma arc melting. This ingot and the other two ingots with the nominal composition of 15W–85Al (at.%) prepared by the same method were melted by vacuum induction melting and poured into the mould. Only a little bit of undissolved W wire remained at the bottom of the melting crucible after this re-melting and casting, and as in the previous induction melting, there were no wires in the casting. EDS analysis (see Figure 3) again revealed significant differences between the bottom and upper parts of the ingot (14 at.% W for the bottom part and 19 at.% for the upper part), but no significant differences were observed with the change of depth from the surface of the mould.

The contents of oxygen and nitrogen in the prepared W–Al alloys were about 180 and 7 wt. ppm, respectively, for W–Al-VIM-1 and about 200 and 10 wt.% ppm, respectively, for W–Al-VIM-2.

### 2.2. Microstructure

#### 2.2.1. Nb–Al Alloys

**Figure 4:**
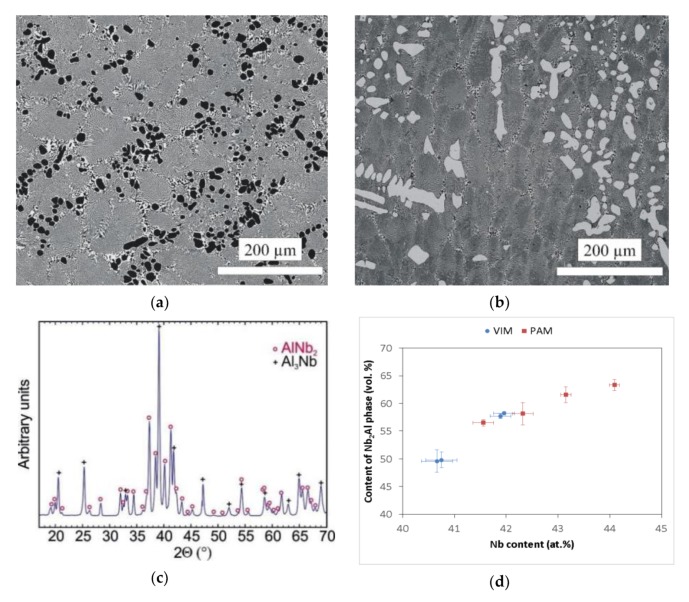
(**a**) SEM-BSE image of the Nb–Al-VIM-1 alloy; (**b**) SEM-BSE image of the Nb–Al-PAM-1 alloy; (**c**) X-ray diffraction patterns of the Nb–Al-VIM-1 alloy; (**d**) dependence between content of Nb_2_Al phase and Nb content in Nb–Al alloys.

The microstructure of the prepared Nb–Al alloys is shown in Figure 4a,b. The structure corresponds, in principle, to the binary diagram [19] and is formed by Nb_2_Al phase (tP30), referred to as ơ phase in the literature [19], which in the micrographs is shown in a light colour, and NbAl_3_ phase (tI8) is shown in a dark colour. The occurrence of Nb_2_Al and NbAl_3_ phases was also confirmed by the X-ray diffraction pattern shown in Figure 4c. However, significant differences in microstructure were observed according to the different contents of Nb in the Nb–Al ingots. The microstructure consisted of coarse particles of NbAl_3_ phase and eutectics formed by the mix of Nb_2_Al and NbAl_3_ phases in the VIM-1 ingot, which was characterized by the lowest content of Nb (40.6–40.8 at.%). This type of microstructure is documented in Figure 4a. Conversely, for all other ingots the microstructure consisted of coarse particles of Nb_2_Al phase and eutectics formed by the mix of Nb_2_Al and NbAl_3_ phases. This type of microstructure is shown in Figure 4b. The microstructures therefore exhibit features of hypereutectic or eutectic alloy for VIM-1, for which primary solidification is NbAl_3_ phase, and features of hypoeutectic alloy for other ingots, for which primary solidification is Nb_2_Al phase. The average contents of elements in individual phases, calculated from the chemical composition determined by EDS analysis, were almost the same in all ingot areas. Niobium content was about 56–57 at.%, 24–26 at.%, and 39–41 at.% for coarse Nb_2_Al phases, coarse NbAl_3_ phases, and eutectics, respectively. However, individual ingots exhibited significant differences in the content of phases identified by automatic image analysis. As can be seen in Figure 4d, the measured content of Nb_2_Al phase was about 50 vol.% in VIM alloys with a Nb content of about 40.6–40.8 at.%, while for VIM hypoeutectic alloys with a Nb content of about 41.9–42.0 at.%, it was higher and reached up to 58 vol.%. The same phenomenon was observed in hypoeutectic PAM alloys, for which the content of Nb_2_Al phase reached values of 56.5–58.2 vol.% for an alloy with 41.6–42.3 at.% Nb to 61.6–63.3 vol.% for an alloy with 43.2–44.1 at.% Nb. This phenomenon was likely caused by different content of coarser Nb_2_Al particles in the hypoeutectic alloy and NbAl_3_ particles in the hypereutectic alloy, because Nb_2_Al phase content in the eutectic reached similar values of about 49–53 vol.% in all alloys, irrespective of the preparation method.

#### 2.2.2. W–Al Alloys

**Figure 5:**
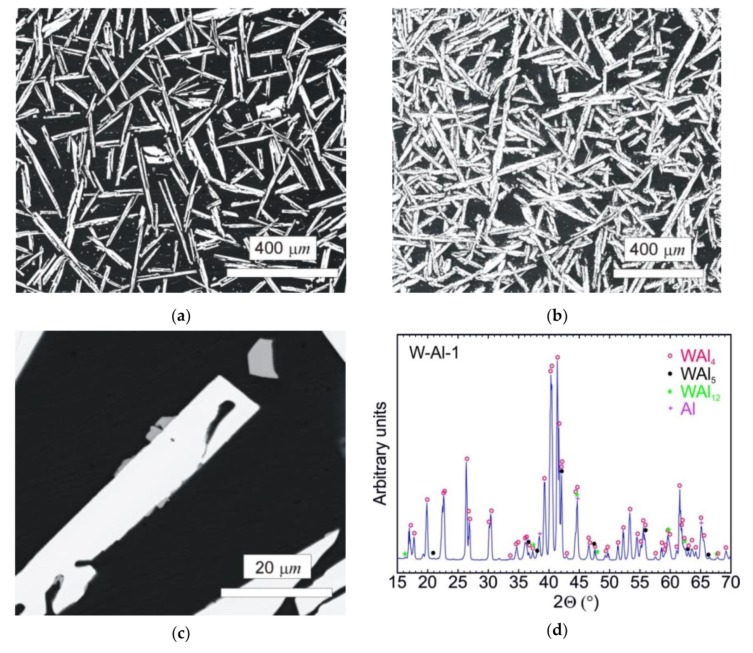
(**a**) SEM-BSE image of the bottom part of the W–Al-VIM-1 alloy; (**b**) SEM-BSE image of the upper part of the W–Al-VIM-1 alloy; (**c**) SEM-BSE image of WAl_12_ phase in the bottom part of the W–Al-VIM-1 alloy; (**d**) diffraction pattern of sample W–Al-VIM-1.

**Figure 6:**
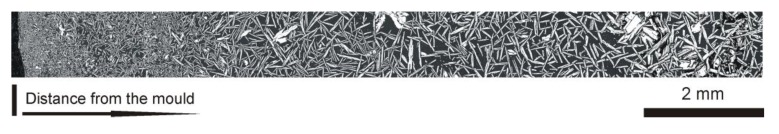
Microstructure evolution with distance from the wall of the mould in the bottom part of the W–Al-VIM-1 ingot.

**Figure 7:**
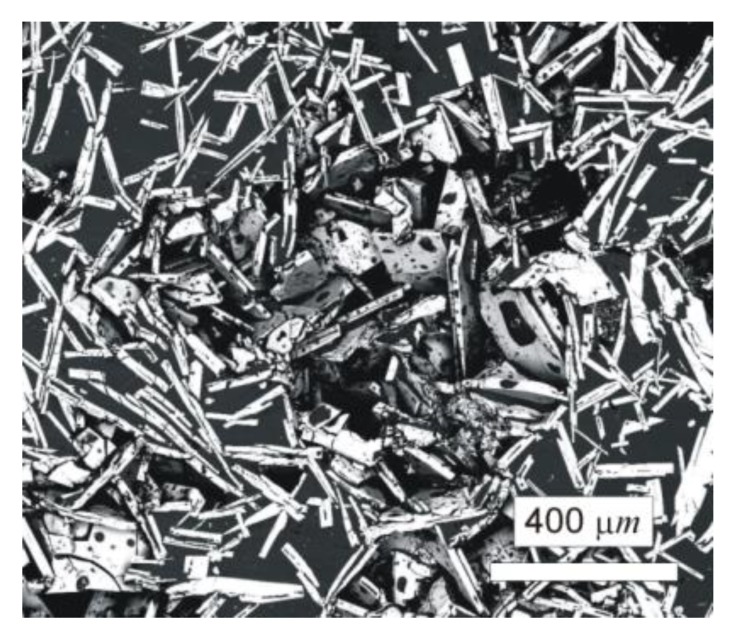
Shrinkage in the upper part of the W–Al-VIM-1 alloy.

The typical microstructure of the W–Al-VIM-1 alloy ingot is shown in Figure 5a,b. The structure did not contain any undissolved W particles and usually consisted of needle-like WAl_4_ particles (mS30), with a chemical composition as measured by EDS between 19 at.% and 25 at.% W. These particles are shown in the attached figures as light phase, whereas the Al matrix (W content up to 0.1 at.%) is shown in black. More detailed microstructure observations at higher magnifications in BSE mode identified another phase, with a darker colour than WAl_4_ phase but lighter than the Al matrix (see Figure 5c). This was identified as WAl_12_ phase (cI26) by EDS analysis of chemical composition (7–10 at.% W), and the presence of this phase in the castings was also confirmed by X-ray diffraction (see Figure 5d). This phase was usually observed at the interfaces between WAl_4_ needles and the Al matrix or as fine particles in the Al matrix. Besides those mentioned, no other phases were observed in the casting. However, the microstructure showed significant differences between the top and bottom of the ingot, and also with the distance from the walls of the mould, as demonstrated in Figure 6 and the results of EDS analysis for individual ingot parts. It is obvious that the upper part of the ingot included a higher proportion of WAl_4_ needle-like phase than the bottom part. The content of WAl_4_ phase in individual sections was 35 vol.% and 58 vol.% for the bottom and upper parts, respectively. Figure 6 shows the typical microstructure evolution with distance from the wall of the mould in the bottom part of W–Al-VIM-1 ingot. The microstructure evinced two distinct zones. The first zone (up to 3 mm from the wall of the mould) was formed by smaller particles (up to 200 µm) of WAl_4_ phase, and their placement in the Al matrix was more regular and dense. The second zone was formed by longer needle-like WAl_4_ particles (up to 1500 µm), and their placement in the Al matrix was irregular and less dense. This difference was probably due to differences in cooling rate, which was higher near the wall of the mould and lower with increasing distance from the wall. This microstructure evolution suggests that with decreasing cooling rate, the WAl_4_ particles were longer and larger, which caused pronounced shrinkage in the central zone of the casting formed by large particles of WAl_4_ phase without Al matrix (see Figure 7). The same shrinkage and microstructure evolution, only with higher proportions of shrinkage, were observed also in the upper part of the ingot. This phenomenon apparently caused an increase of W content in the central part of the casting, which was identified during EDS analysis, as shown in Figure 3.

**Figure 8:**
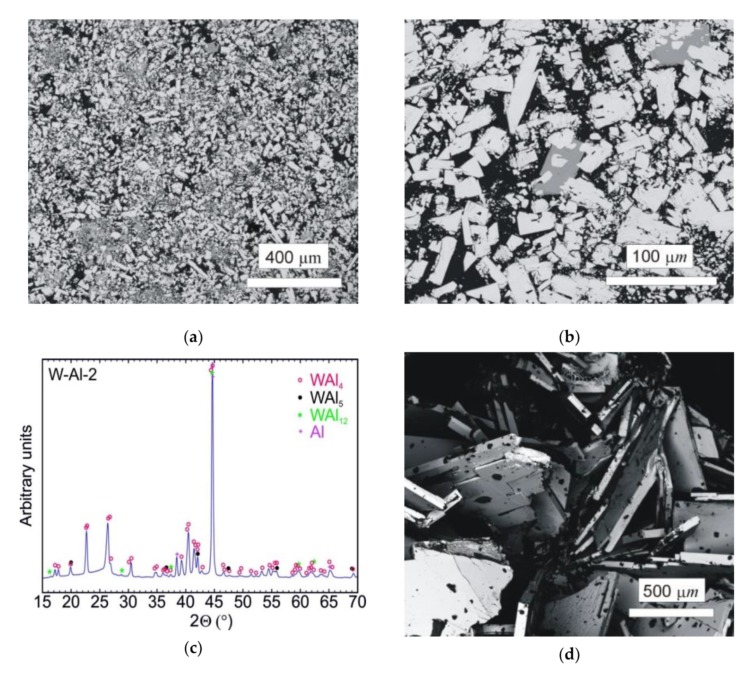
(**a**) SEM-BSE image of the bottom part of the W–Al-VIM-2 alloy; (**b**) SEM-BSE image of WAl_12_ phase in the bottom part of the W-Al-VIM-2 alloy; (**c**) diffraction pattern of sample W–Al-VIM-2; (**d**) SEM-BSE image of the upper part of the W–Al-VIM-2 alloy.

**Figure 9:**
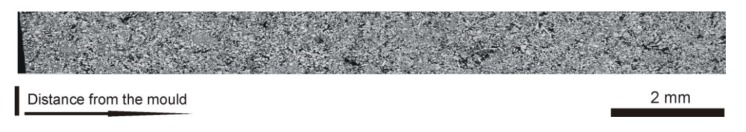
Microstructure evolution with distance from the wall of the mould in bottom part of W–Al-VIM-2 ingot.

The typical microstructure of the W–Al-VIM-2 alloy ingot is shown in Figure 8a,b. No undissolved W particles were observed, and the structure consisted of WAl_4_ particles, WAl_12_ particles, and Al matrix. The chemical composition of these phases showed no changes in comparison with the phases in the W–Al-VIM-1 ingot, and the W content was about 22, 8 and 0.5 at.% for WAl_4_ phase, WAl_12_ phase, and Al matrix, respectively. The presence of these phases was also confirmed by XRD patterns, as shown in Figure 8c. XRD patterns also contained a small amount of WAl_5_ phase, but this phase was not found in the microstructure. Significant differences in shape, size, and amount of WAl_4_ particles were found. The WAl_4_ particles were smaller (up to 200 µm), did not exhibit a typical needle-like shape, and their content in the bottom part was about 67 vol.%, which is approximately twice that in the bottom part of W–Al-VIM-1 (35 vol.%). As can be seen in Figure 9, the microstructure in the bottom part of W–Al-VIM-2 alloy did not show two distinct zones as in W–Al-VIM-1, and the microstructure was nearly homogenous throughout the volume of this part. However, the structure of the upper part of the W–Al-VIM-2 alloy was completely different and consisted of only large WAl_4_ particles (up to 2000 µm) without or with a small amount (up to 5%) of Al matrix. A typical example of this microstructure is documented in Figure 8d.

### 2.3. Melting Temperatures and Phase Transformations

#### 2.3.1. Nb–Al Alloys

**Figure 10:**
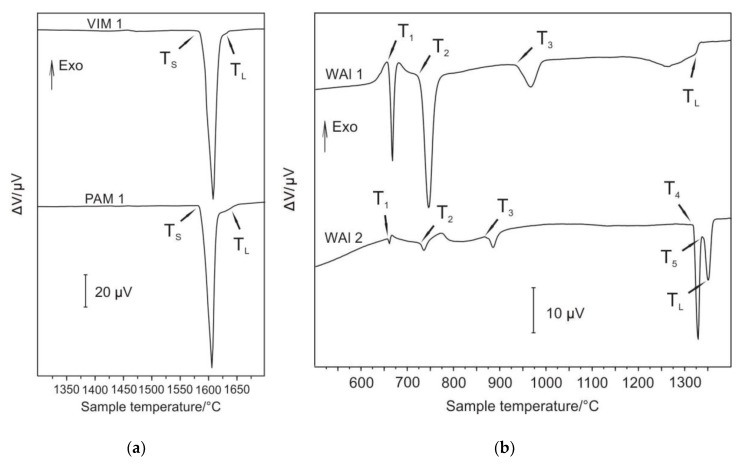
(**a**) Differential thermal analysis (DTA) curves for samples Nb–Al-VIM-1 and Nb–Al-PAM-1; (**b**) DTA curves for samples W–Al-VIM-1 and W–Al-VIM-2.

The selected samples were subjected to differential thermal analysis (DTA) in order to determine important phase transformations and liquidus temperature, mainly due to characterisation of microstructure stability at higher temperatures and also to confirm the course of solidification. For the measurements, the bottoms of samples Nb–Al-VIM-1 and Nb-Al-PAM-1 were selected, of which Nb content was 40.8 at.% and 44.1 at.%, respectively. The chemical composition of all individual samples used for DTA was determined before measurement by EDS analysis. The result of this method is a DTA curve. It is possible to determine from obtained DTA curves, based on the peaks revealed, the running thermal events at heating (and cooling, if needed) and consequent phase transition temperatures. These curves were obtained and evaluated with the use of SW SETSOFT. Figure 10a shows DTA curves of samples obtained at a heating run at the rate of 15 °C/min with marking of characteristic temperatures of phase transformations. DTA curves showed a similar shape for both measured samples. Two endothermic heat effects (the first significantly large and the second very small) were observed, corresponding to the eutectic transformation, followed by further melting (dissolution) of Nb_2_Al (PAM-1 bottom) and NbAl_3_ (VIM-1 bottom). As the alloys were very close to the eutectic point, they transformed predominantly eutectically. Solidus (T_S_) and liquidus (T_L_) temperatures were obtained and are denoted in Figure 10a and Table 3. The determined temperatures of solidus and liquidus of the VIM-1 alloy were 1583 °C and 1622 °C, respectively, and those of the PAM-1 alloy were 1583 °C and 1626 °C, respectively. Other than the noted peaks, DTA curves exhibited no further heat effects, indicating that at lower temperatures no significant phase transformations took place. The solidification of Nb–Al alloys can be described based on DTA and results obtained as follows:

L = L + Nb_2_Al = Nb_2_Al + NbAl_3_, (1)

L = L + NbAl_3_ = Nb_2_Al + NbAl_3._ (2)

**Figure 11:**
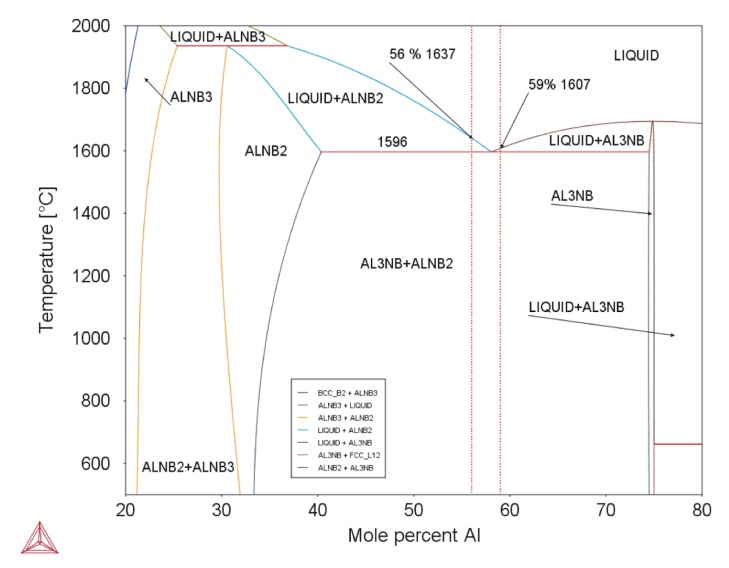
Binary diagram of Nb–Al system (Thermo-Calc; dotted red lines mark composition of analysed alloys).

Equation (1) is valid for Nb–Al-PAM-1 and Equation (2) for Nb–Al-VIM-1. The eutectic temperature was determined as 1583 °C by DTA, and the eutectic composition was revealed by microstructural examination as being between 58.4 at.% and 59.3 at.% Al. This temperature and composition are in good agreement with the binary diagram calculated in the Thermo-Calc software (see Figure 11) and with the results reported by Loser, who reported a eutectic composition of 59–60 at.% Al [8].

#### 2.3.2. W–Al Alloys

For DTA measurements, the samples of the bottoms of W–Al-VIM-1 and W–Al-VIM-2 were selected, of which W content was 7.1 at.% and 14.2 at.%. More phase transitions were observed in W–A1 alloys. The DTA curve (Figure 10b) for the WAl-1 sample showed four heat effects corresponding to phase transitions, and for the WAl-2 sample, five heat effects. Characteristic phase transition temperatures were evaluated for WAl-1 (T_1_, T_2_, T_3_, and T_L_) and WAl-2 (T_1_, T_2_, T_3_, T_4_, T_5_, and T_L_); T_1_ and T_2_ correspond to each other (see Table 4). Temperature T_3_ differs by about 77 °C. The melting process was finished by WAl-1 at T_L_ = 1314 °C. The transition of WAl_4_ to W_23_Al_77_ occurred at 1316 °C (T_4_, WAl-2). Another isothermal phase transition was observed at 1334 °C (see Table 4). The melting process was finished (WAl-2) at liquidus temperature T_L_ = 1340 °C. Solidification of W–Al alloys can be described based on DTA and results obtained as follows:

L = L + W_23_Al_77_ = L + WAl_4_ = L + WAl_4_ + WAl_5_ = WAl_4_ + WAl_5_ + WAl_12_ + Al, (3)

L = L + WAl_4_ = L + WAl_4_ + WAl_5_ = WAl_4_ + WAl_5_ + WAl_12_ + Al. (4)

**Figure 12:**
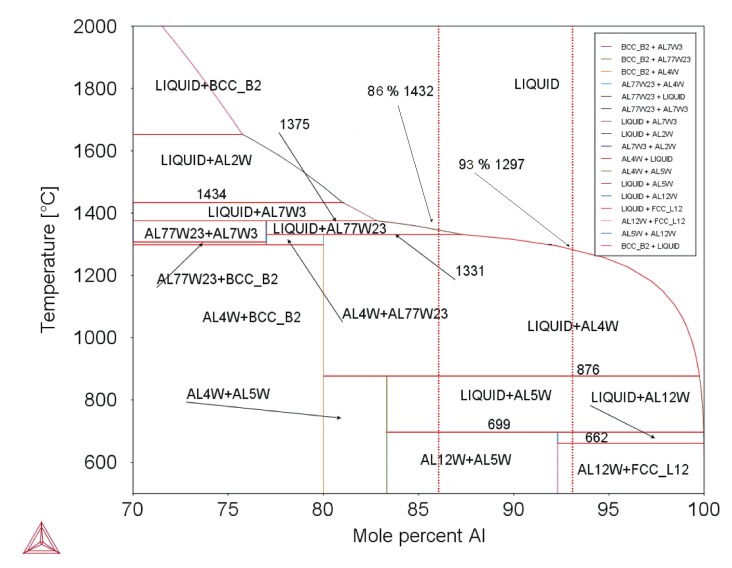
Binary diagram of W–Al system (Thermo-Calc; dotted red lines mark composition of analysed alloys).

Equation (3) is valid for W–Al-VIM-2 and Equation (4) for W–Al-VIM-1. The binary diagram (see Figure 12) shows that W–Al-VIM-2 should contain only WAl_5_ and WAl_12_ phases and W–Al-VIM-1 only WAl_12_ phase and Al at room temperature. However, this would only be achieved at a very low cooling speed. Fast cooling (cold mould) was used for both VIM and PAM, which did not allow thermodynamic equilibrium. This is why Al and WA1_4_ phase needles were observed in W–Al alloys.

## 3. Materials and Methods

Commercially available metals (purity 99.9%) were cut into 10 × 20 × 40 mm Al plates, 1 × 10 mm W wires, and 2 × 10 mm Nb wires, and were used as input materials for melting. Two melting methods with different heating principles and crucible materials were used to prepare the alloys. The first method was vacuum induction melting (VIM) with the use of a commercially available corundum crucible under a vacuum of 5 Pa. The furnace was a Leybold IS3/1 with an output power of 30 kW. The placement of charges in the crucible before melting was nearly identical for both alloys. Charges were placed in the crucible as follows: half of the Al plates in the bottom of the crucible, Nb or W wires in the middle, and the second half of Al plates in the upper part. After the evacuation, the power of the induction furnace was gradually increased and the first Al plates started to melt. When the first liquid metal was observed, the furnace was filled with commercially pure dry argon (purity 99.95%) to a pressure of 20–25 kPa and power was constantly increased. After stabilization at a temperature of about 1600 °C (measured by optical pyrometer) for 60 and 120 s (for Nb–Al alloy) and 240 s (for W–Al alloy), the melt was cast into a cold copper mould. The second method was plasma arc zone melting (PAM) with the use of a horizontal water-cooled copper mould. The plasma furnace was equipped with a plasma nozzle 16 mm wide with a 50 mm arc length and worked under a voltage of 63 V. A more detailed description of this device is given in [20]. The placement of the charge in the crystallizer was the same as for vacuum induction melting, with the wire of the refractory metals placed in the centre and pieces of Al in the bottom and upper part of the crystallizer in the vertical direction. After evacuation and flushing by Ar (purity 99.95%), the plasma torch was started and melting was done in a flowing Ar atmosphere (27 L/min) with a maximum current density of 800 A. After every 2 re-meltings (second melting always in the opposite direction to the first), the ingots were rotated 180° in the vertical direction. The melting process was conducted 2 and 4 times, with a crystallizer feed rate of 0.5 cm/min for Nb–Al alloys and 4, 6, and 8 times with a crystallizer feed rate of 0.3 cm/min for W–Al alloys.

Samples for metallographic investigation were cut from the as-cast alloys by electro-erosion cutting (cutting in running water at a voltage of 1.8 kV and a current of 150 mA). Standard metallographic techniques, including grinding on SiC papers with grain sizes ranging from 60 to 2000 (grains/cm^2^) and polishing with Al_2_O_3_ suspension with particle size changing from 1 to 0.3 μm, were applied. The samples were studied by optical microscopy (OM) and scanning electron microscopy in back-scattered electron (BSE) mode using a Quanta 450 FEG microscope (FEI Company, Fremont, CA, USA) equipped with an energy-dispersive X-ray spectrometer (EDS). EDS analysis was performed on 2 transverse sections taken from the bottom and upper part (below the feeding head after vacuum induction melting and where the plasma torch was stopped during plasma melting) of each cast bar (see Figure 1 and Figure 2). The average chemical composition measured on the specific transverse sections of the bars was calculated from 8 measurements, with an arrangement from the area near the surface to the centre of the induction-melted ingots and from the surface of the bottom side of the transverse section (with contact with a water-cooled mould) to the second surface in the vertical direction. The average chemical composition of identified phases was calculated from a minimum of 5 spot measurements made in different phase areas by EDS. Volume fractions of coexisting phases were determined from digitalized BSE micrographs using a Fiji ImageJ computer image analyser (Madison, WI, USA). XRD analysis was carried out using a Bruker D8 DISCOVER diffractometer (Billerica, MA, USA) equipped with an X-ray tube with rotating Cu anode operating at 12 kW. All measurements were performed in parallel beam geometry with a parabolic Goebel mirror in the primary beam. Diffraction patterns were measured within an angular range of 20–70° of 2Ɵ with an exposition time of 5 s and step size of 0.05°. The oxygen content was measured by the thermo-evolution method on an ELTRA ONH–2000 instrument (Haan, Germany). At least 3 pieces from each sample were analysed. Differential thermal analysis (DTA, based on the measurement of temperature difference between sample and reference) was performed with the use of Setaram SETSYS 18TM experimental equipment (SETARAM Instrumentation, Caluire, France). The chemical composition of all individual samples used for DTA was determined before the measurement by EDS analysis. Samples with dimensions of 3 × 3 × 3 mm were prepared by electro-erosion cutting and wet grinding on 600 grit sandpaper. Samples were analysed in corundum crucibles under a high-purity inert atmosphere of Ar (6N). Before analysis, the inner space of the furnace was flushed by inert gas and then evacuated 3 times and filled with Ar. DTA was performed during the heating and cooling process (15 °C/min). Phase transition temperatures were evaluated from DTA curves obtained in the heating run. Temperature calibration was performed with respect to the melting points of Pd (5N) and Ag (5N). Correction of phase transition temperatures was performed with respect to the sample mass and heating rate. For the calculation of phase diagram cuts, Thermo-Calc software (version 2015b, Solna, Sweden) and SSOL5–SGTE Solution Database version 5 (Solna, Sweden)(thermodynamic database containing critical assessments of many binary and ternary and some higher-order systems), were used. Thermo-Calc uses a Calphad approach for all thermodynamic calculations. Default settings of phases and species were used for thermodynamic calculations of phase diagrams. More specific information about the software and database used can be found in [21].

## 4. Conclusions

Metallurgical preparation of Nb–Al and W–Al intermetallic compounds and the characterization of their microstructure and phase transformations by DTA were investigated. The achieved results can be summarized as follows:

(1) Significant evaporation of Al was observed during all melting processes, which affected the resulting chemical composition.
(2) Alloys of 40Nb–60Al (at.%) with a weight of 420 g were prepared by VIM at a temperature of 1600 °C with a stabilization time of 60 and 120 s. The alloys exhibited homogeneous composition and low levels of O and N.
(3) Alloys of 40Nb–60Al with a weight of 160 g were prepared by PAM. The prepared alloys exhibited inhomogeneous composition (difference of about 1% between the bottom and top of the ingot), which was not removed by further re-melts.
(4) PAM did not allow preparation of intermetallic compounds based on W–Al because the microstructure showed residues of non-molten W parts in the castings.
(5) VIM allowed the preparation of a W–Al intermetallic compounds with a weight of 420 g, a W content up to 10 at.% and without non-melted W parts. However, the chemical composition at the bottom and top of the ingot showed a significant difference up to 2 at.%.
(6) The combination of PAM and VIM allowed the preparation of W–Al intermetallic compounds with a weight of 420 g, containing W up to 19 at.%. However, the chemical composition at the bottom and top of the ingot showed a significant difference of up to 5 at.%.
(7) The microstructure of Nb–Al alloys consisted of Nb_2_Al and Nb_2_Al, NbAl_3_ eutectic for compounds with Nb content up to 41 at.%. The compound with a lower content of Nb consisted of NbAl_3_ phase and Nb_2_Al, NbAl_3_ eutectic.
(8) The microstructure of W–Al alloys consisted of WAl_4_ and WAl_12_ phases in Al matrix.
(9) The size, shape, and content of the WA1_4_ phases in the structure exhibited significant differences depending on the cooling rate after casting.

## Figures and Tables

**Table 1:** List of all prepared alloys.

Alloy	Nominal Composition (at.%)	Method Used	Number of Passes	Melting Temperature (°C)/Stabilization Time (s)	Weight of Charge (g)
Nb–Al-VIM-1	40Nb-60Al	Vacuum induction melting	–	1600/60	420
Nb–Al-VIM-2	40Nb-60Al	Vacuum induction melting	–	1600/120	420
Nb–Al-PAM-1	40Nb-60Al	Plasma arc melting	4	–	160
Nb–Al-PAM-2	40Nb-60Al	Plasma arc melting	2	–	160
W–Al-VIM-1	15W-85Al	Vacuum induction melting	–	1600/240	420
W–Al-PAM-1	15W-85Al	Plasma arc melting	4	–	150
W–Al-PAM-2	15W-85Al	Plasma arc melting	6	–	150
W–Al-PAM-3	15W-85Al	Plasma arc melting	8	–	150
W–Al-VIM-2	15W-85Al	Plasma arc melting + vacuum induction melting	6	1500/30	420

**Table 2:** Determined chemical composition in individual areas by EDS.

Area	Content of W (at.%)
1	99.4 ± 0.5
2	9.7 ± 0.4
3	19.9 ± 0.5
4	0.15 ± 0.06

**Table 3:** Measured values of liquidus and solidus in Nb–Al-VIM-1 and Nb–Al-PAM-1 samples by DTA and detected values with using Thermo-Calc software (all results in °C).

	T_S_		T_L_	
Sample	DTA	Thermo-Calc	DTA	Thermo-Calc
Nb–Al-VIM-1	1583	1596	1622	1607
Nb–Al-PAM-1	1583	1596	1626	1637

**Table 4:** Measured values of phase transformations in W–Al-VIM-1 and W–Al-VIM-2 samples by DTA and detected values using Thermo-Calc software (all results in °C).

Sample	Al_12_W + Al = L + Al_12_W		L + Al_12_W = L + Al_5_W		L + Al_5_W = L + Al_4_W		L + Al_4_W = L + Al_77_W_23_		T_L_	
	DTA	Thermo -Calc	DTA	Thermo -Calc	DTA	Thermo -Calc	DTA	Thermo -Calc	DTA	Thermo -Calc
**WAl-1**	656	662	715	699	931	876	–	1331	1324	1305
**WAl-2**	656		721		868		1319		1350	1418
